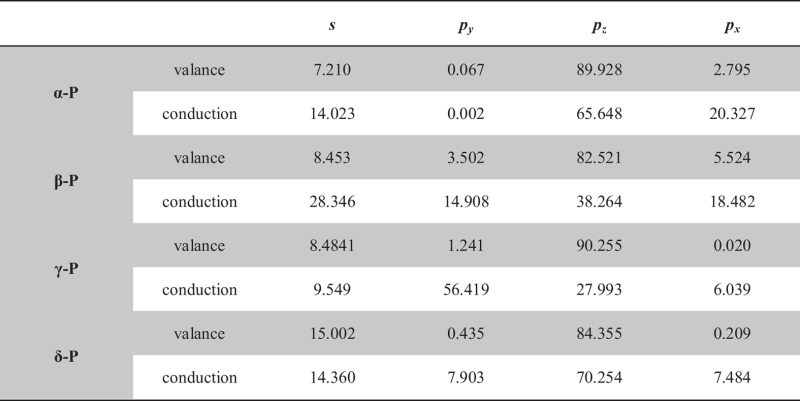# Theoretical predictions on the electronic structure and charge carrier mobility in 2D Phosphorus sheets

**DOI:** 10.1038/srep09961

**Published:** 2015-06-02

**Authors:** Jin Xiao, Mengqiu Long, Xiaojiao Zhang, Jun Ouyang, Hui Xu, Yongli Gao

**Affiliations:** 1 Institute of Super-microstructure and Ultrafast Process in Advanced Materials, School of Physics and Electronics, Central South University, Changsha 410083, China; 2 Department of Physics and Materials Science, City University of Hong Kong, Hong Kong, China; 3 Department of Physics and Astronomy, University of Rochester, Rochester, NY 14627, USA

## Abstract

We have investigated the electronic structure and carrier mobility of four types of phosphorous monolayer sheet (α-P, β-P,γ-P and δ-P) using density functional theory combined with Boltzmann transport method and relaxation time approximation. It is shown that α-P, β-P and γ-P are indirect gap semiconductors, while δ-P is a direct one. All four sheets have ultrahigh carrier mobility and show anisotropy in-plane. The highest mobility value is ~3 × 10^5^ cm^2^V^−1^s^−1^, which is comparable to that of graphene. Because of the huge difference between the hole and electron mobilities, α-P, γ-P and δ-P sheets can be considered as *n*-type semiconductors, and β-P sheet can be considered as a *p*-type semiconductor. Our results suggest that phosphorous monolayer sheets can be considered as a new type of two dimensional materials for applications in optoelectronics and nanoelectronic devices.

Since the successful preparation of graphene^1^, two-dimensional(2D) atomically-thick materials, such as graphdiyne sheet^2^, boron nitride sheet^3^, silicene^4^ and layered transition-metal dichalcogenides^5^,^6^ have attracted intensive attention owing to their unique physical properties and potential applications in nanoscale devices. Recently, due to the synthesis of few layers black phosphorus (BP)^7^,^8^,^9^,^10^, named phosphorene, 2D phosphorous materials have become the focus of science community^11^,^12^,^13^,^14^,^15^,^16^,^17^,^18^,^19^,^20^,^21^,^22^. BP is the most stable phosphorus allotrope under normal conditions with a direct band gap of about 0.3 eV^23^,^24^,^25^. The direct band gap will increase to ~2.0 eV^26^,^27^ as BP reduces to a monolayer, which opens doors for applications in optoelectronics. Furthermore, bulk BP is found to have high carrier mobility in the order of 10^5^ cm^2^V^−1^s^−1^ at low temperatures^28^,^29^. The field-effect carrier mobility of few-layer BP is measured to be still higher, up to 1000 cm^2^V^−1^s^−1^ for electron^7^ and 286 cm^2^V^−1^s^−1^ for hole^8^ at room temperature. Also, few-layer BP exhibits ambipolar behavior with drain current modulation up to 10^5^^7^. Owing to the direct gap and high mobility, there is a high potential for BP thin crystals to be a new 2D material for applications in optoelectronics, nanoelectronic devices and so on^30^,^31^,^32^,^33^,^34^,^35^,^36^.

So far, there have been few reports about the mobility of monolayer BP experiment researches. Theory studies based on effective mass calculation^25^ have shown that the room temperature electron mobility of monolayer BP is over 2000 cm^2^V^−1^s^−1^^11^ or 5000 cm^2^V^−1^s^−1^^19^. However, the method is subject to the parabolic properties of the energy bands. In this paper, both of electron and hole mobilities are investigated with Boltzmann transport equation (BTE) method beyond the effective mass approximation. Furthermore, besides the monolayer BP (marked as α-P), other three stable 2D phosphorus allotropes, namely β-P, γ-P and δ-P based on theoretical predictions^20^,^21^, have also been investigated. We therefore report the first theoretical prediction on the charge mobility of those 2D phosphorus allotropes in this work.

## Results

**Figure 1:**
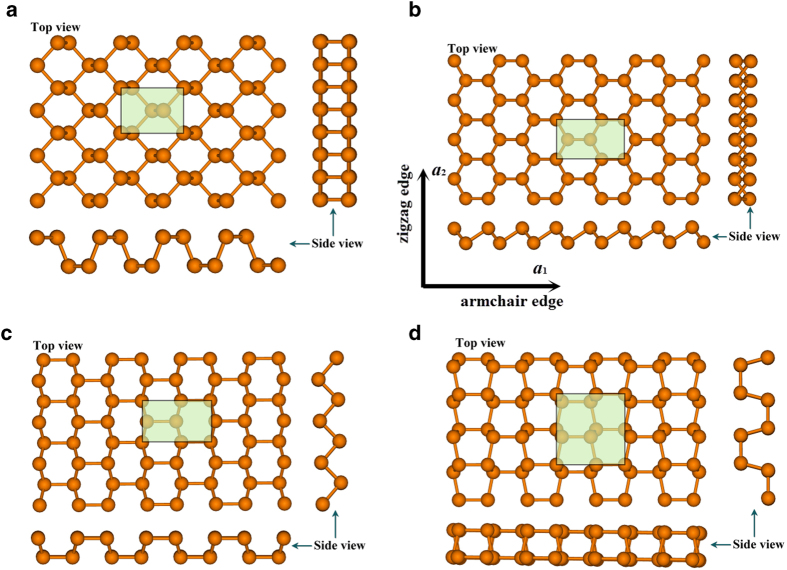
The models of phosphorus sheets: (**a**) α-P, (**b**) β-P, (**c**) γ-P and (**d**) δ-P.

The atomic structures of four different types of phosphorus sheets are shown in Fig. 1. In order to get an intuitive demonstration of carrier conduction along the armchair and zigzag directions, an orthogonal supercell covered by a green shadow is used in Fig. 1. The lattice length is shown in Table 1, and is in agreement with previous studies. There are four phosphorus (P) atoms in the supercell of α, β, γ phosphorus (α-P, β-P, γ-P), and eight P atoms in the supercell of δ phosphorus (δ-P). There are two phosphorus sub-layers in each phosphorus sheet. The distance of two P sub-layers (d) is shown in Table 1. The energy per atom indicates that α-P sheet is the most stable. The average P bond length is about 2.23 ~ 2.27 Å.

**Figure 2:**
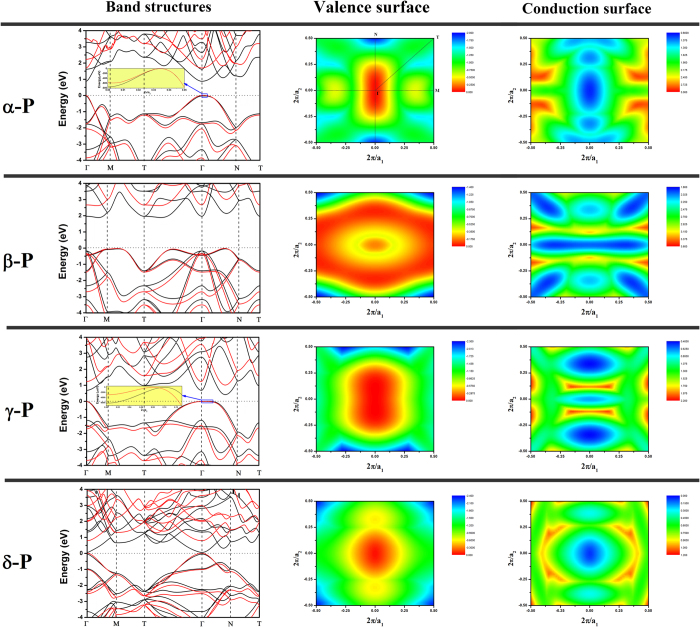
Energy band structures, valance band edge surface heat map and conduction band edge surface heat map of α-P, β-P, γ-P and δ-P. Red arrows are the direct gaps at Γ point. The black lines and red lines in band structures are calculated by PBE and HSE06 respectively. K point: Γ(0,0,0), Μ(0.5, 0, 0), Ν (0, 0.5, 0), Τ(0.5, 0.5, 0).

Energy band structures and Fermi surface heat map of the phosphorus sheets are shown in Fig. 2. All four types of phosphorus sheets are semiconductors. For α-P, β-P, γ-P and δ-P, as shown in Table 2, the energy band gaps based on PBE (HSE06) calculation are 0.91(1.70), 1.93(2.64), 0.42(1.03) and 0.10(0.78) eV, respectively. The energy band structures and band gaps are consistent to those reported in previous studies^21^. Our calculations indicate that only δ-P is a direct gap semiconductor. The other three type sheets are indirect semiconductors. These results are in good agreement with previous study^20^,^21^. Interestingly, when we zoom in the energy band spectrum around the Γ point in α-P, as shown in the inset of Fig. 2 we can find that the top of the valence band is located at (0, 0.035) K point (skewed slightly along the zigzag direction) and is about 0.75 (0.53) meV higher than the Γ point based on the PBE (HSE06) calculation. This tiny skewing away Γ point on the top of the valence band is in good agreement with Ref. 37 and has also been demonstrated in α-P zigzag nanoribbon^16^. The optical characteristics should be influenced only slightly in α-P sheets due to such tiny skewing.

Based on the band structures, we calculate the effective mass of the charge carrier by parabolic fitting near the Fermi surface, which is presented in Table 3. It can be found that most of |m*| is smaller than the mass of the free electron (*m*_*e*_), which means that the phosphorus sheets have considerably high carrier mobility. Our results show that the m* for electrons and holes in α-P are 0.1382 and 1.2366 *m*_*e*_, respectively, which are in good agreement with *Yang’s* report^11^. Furthermore, it is clearly seen that the |m*| of electron or hole along the armchair direction over an order of magnitude smaller than that along the zigzag direction. It indicates that the carrier transport is anisotropic and the armchair direction is the main transport direction in α-P. The case in β-P is the opposite. The |m*|of electron or hole along the zigzag direction is three times larger than that along the armchair direction, which means that the carrier transport ability is stronger along the armchair than the zigzag direction in β-P. It is easily to see that the |m*| of hole along the zigzag direction in α-P and γ-P is much larger than others’, which result from the almost flat valence band in those materials.

**Figure 3:**
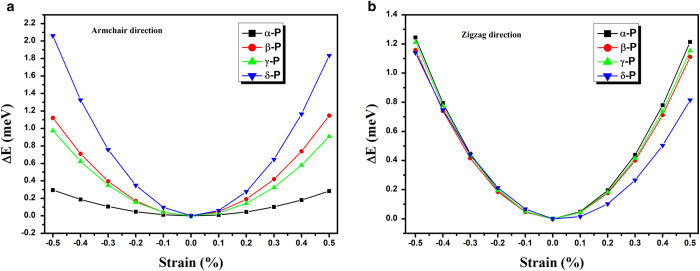
Energy−strain relationship along armchair (**a**) and zigzag (**b**) directions.

The variation of total energy (*E*) with uniaxial strain (*δ*) applied along the armchair and zigzag directions are shown in Fig. 3. Based on those energy-strain curves, the in-plane stretching modulus *C*^*2D*^ can be obtained. In α-P sheets, we can also find that *C*^*2D*^ is obvious anisotropic, and it is about four times larger along the zigzag direction (103.278 N/m) than along the armchair direction (24.255 N/m). These are in good agreement with *Qiao*’s report (101.60 and 28.94 N/m)^25^. In general, the three-dimensional Young’s modulus can be estimated as *C*^*3D*^ = *C*^*2D*^/*t*_*0*_. Based on the optB86b van der Waals functional, the interlayer separation of α-P, β-P, γ-P and δ-P have been calculated as 5.30, 4.20, 4.21 and 5.47 Å^21^, respectively. By assuming a finite thickness (*t*_*0*_ = 5.30, 4.20, 4.21 and 5.47 Å) for α-P, β-P, γ-P and δ-P sheet, the Young’s modulus along the armchair and zigzag direction are shown in Table 4. The previous theoretical study has shown the Young’s modulus of monolayer α-P sheet to be 44 GPa (armchair direction) and 166 GPa (zigzag direction)^31^.

**Figure 4:**
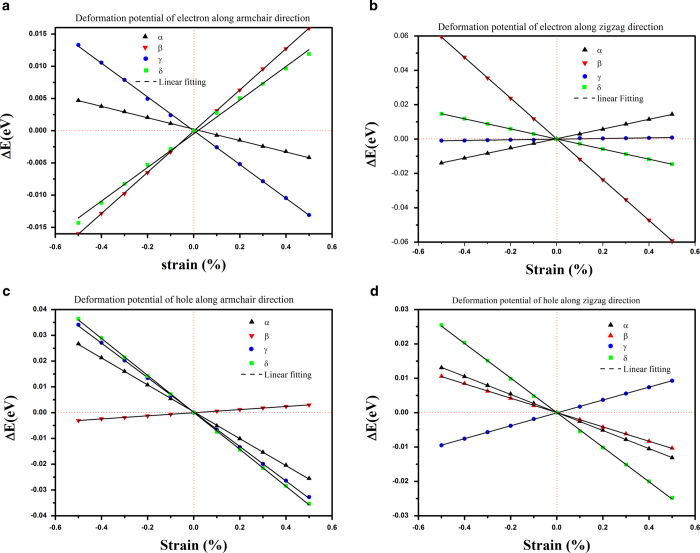
Shifts of conduction band and valence band under uniaxial strain: conduction band along (**a**) armchair and (**b**) zigzag direction; valence band along (**c**) armchair and (**d**) zigzag direction. The balck dashed line is the linear fitting.

Figure 4 shows the shifts of band edges as a function of strain along the armchair and zigzag directions. Through dilating the lattice along the armchair and zigzag directions, the DP constant *E*_*1*_ is then calculated as *dE*_*edge*_/*dδ*, equivalent to the slope of the fitting lines, where *E*_*edge*_ is the energy of the conduction (valence) band edge. Each line is fitted by 11 points. The *E*_*1*_ values of phosphorus sheets are shown in Table 5. The standard error of all *E*_*1*_ values is smaller than 1% excluding three valuses marked in Table 5.

On the basis of our energy band spectrum, we calculated *E*_*1*_ and *C*^*2D*^, the acoustic phonon-limited mobility (using Eq. 1) and relaxation time (using Eq. 2) at room temperature (300 K). The results are shown in Table 5. It can be seen that the electron relaxation time (*τ*_*e*_) in phosphorus sheets is much longer than the hole relaxation time (*τ*_*h*_), excluding β-P. The electron mobilities of α-P, β-P, γ-P and δ-P sheets are about 1.1 × 10^4^, 4.7 × 10^2^, 2.9 × 10^5^ and 3.0 × 10^3^ cm^2^V^−1^s^−1^, respectively. The corresponding *τ*_*e*_’s are about 1.29, 0.09, 70.73 and 0.61 ps. The hole mobilities of α-P, β-P, γ-P and δ-P sheets are about 2.0 × 10^2^, 1.7 × 10^3^, 7.3 × 10^1^ and 5.9 × 10^2^  cm^2^V^−1^s^−1^, respectively. The corresponding *τ*_*h*_’s are about 0.02, 0.86, 0.24 and 0.09 ps. It can be found that all four phases have higher mobility than MoS_2_ monolayer sheet (the hole mobility is 86 cm^2^V^−1^s^−1^ and electron mobility is 44 cm^2^V^−1^s^−1^)^38^. Due to the very small conduction band deformation potential (0.187 eV), the electron mobility along the zigzag direction in γ-P sheet is as high as ~3 × 10^5^ cm^2^/Vs, which is in the same order of magnitude of that in graphene^42^,^47^, silicone^39^ and germanene^40^. The minimum is the electron mobility along the zigzag direction in β-P sheet, which is about 47.32 cm^2^/Vs. The electron carriers move faster than the hole ones in α-P, γ-P and δ-P sheet. Only in β-P sheet, the hole mobility is higher than the electron mobility. γ-P sheet has the best electron carrier transmitting capacity and the biggest difference between the electron and hole mobility in four type sheets. Moreover, the obvious anisotropy in carrier mobility can be found. The charge carriers move faster along the armchair direction than the zigzag direction in α-P, β-P and δ-P sheet. While in γ-P sheet, the zigzag direction is preferred.

## Discussion

It must be noted that the mobility in our calculation is a theoretical value. Only the acoustic phonon scattering mechanism is considered. Actually, there are inevitably impurities and defects in the vast majority of materials, and they have a great influence on the charge transport properties, especially at low temperatures where phonon has little effect^47^. For example, in a MoS_2_ sheet, owing to scattering from charged impurities, the mobility at low-temperatures signally decreases with temperature^41^. So the mobility of phosphorus sheets measured experimentally can be much smaller than theoretically predicted.

**Figure 5:**
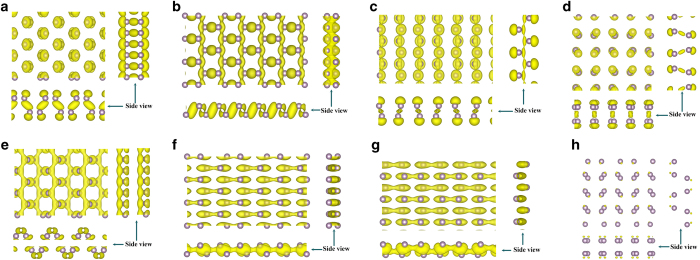
Band decomposed charge density of phosphorus sheets: (**a**)–(**d**) is the valance band edge for α-P, β-P, γ-P and δ-P respectively; (**e**)–(**h**) is the conduction band edge for α-P, β-P, γ-P and δ-P respectively. The isosurface value is 0.01. Drawings are produced by VESTA software^42^.

The band decomposed charge density around the Fermi level of phosphorus sheets is shown in Fig. 5. The composition of the top valence and the bottom conduction band are shown in Table 6. Atomic orbital analysis shows that the top of valence states in phosphorus sheets are mainly composed of 3*p*_*z*_ orbits. Our calculations indicate that, in α-P, the conduction bands are mainly composed of *p*_*z*_ orbitals with mixed *s* and *p*_*x*_ orbits. In β-P, the conduction bands are hybridization orbitals with mixed *s* and *p* orbits. In γ-P, the conduction bands are mainly composed of *p*_*y*_ and *p*_*z*_ orbitals. In δ-P, the conduction bands are major composed of *p*_*z*_ and *s* orbitals.

In α-P, β-P and γ-P, owing to the valence bands partly composed of in-plane *p* orbits, the top of the valence band is skewing awaly the Γ point. Due to the distribution of the mainly charge density of the valence is along the armchair direction, the hole carrier will move faster along the armchair direction than the zigzag direction in α-P, β-P and δ-P. While in γ-P, due to the contributions of *s* and *p*_*z*_ orbitals, the distribution of valence band charge density is along the zigzag direction (Fig. 5c). So the hole mobility in γ-P is slightly higher along the zigzag direction than the armchair direction. For the conduction band, the distribution of charge density is along the armchair direction, as shown in Fig. 5e,f, and g. This is identical with the electron mobility except γ-P. The orbital analysis shows that the proportion of *p*_*x*_ orbital is larger than that of *p*_*y*_ orbit except γ-P and δ-P. Due to the contribution of *p*_*x*_ orbitals, the electron mobility is higher along the armchair direction than the zigzag direction in α-P and β-P. In γ-P, the contribution of *p*_*y*_ orbits is over 50%. At the same time, there is much lower deformation potential. So the electron mobility along zigzag is surperisely high in γ-P.

## Conclusions

In summarily, we have calculated the electronic structures and the intrinsic charge carrier mobility of four type phosphorus sheets (α-P, β-P, γ-P and δ-P), using first-principles density functional theory and the BTE with the relaxation time approximation. We find that α-P, β-P and γ-P are indirect gap semiconductors. The numerical results indicate that the electron mobility of α-P, γ-P and δ-P sheets at room temperature (about 1.107 × 10^4^, 2.895 × 10^5^ and 3.022 × 10^3^ cm^2^V^−1^s^−1^, respectively) is much higher thanthe corresponding hole mobility (about 204.288, 72.645 and 586.339 cm^2^V^−1^s^−1^, respectively). Nevertheless, in β-P sheet, the hole mobility (1.711 × 10^3^ cm^2^V^−1^s^−1^) is about four times of electron mobility (466.262 cm^2^V^−1^s^−1^). Owing to the huge difference mobilities in hole and electron, α-P, γ-P and δ-P sheets can be considered as *n*-type semiconductors, and β-P sheet can be considered as *p*-type semiconductors. All four types of phosphorus sheets present anisotropy in carrier mobility. Charge carriers move faster along the armchair direction than the zigzag direction in α-P, β-P and δ-P sheet. But in γ-P sheet, the more favorable charge transmission direction is along zigzag.

## Methods

In this paper, the carrier mobility is calculated by BTE method beyond the effective mass approximation which is used to predict the mobility of semiconductor nanometerials, like graphene, carbon nanotubes and so on^2^,^43^,^44^,^45^,^46^,^47^,^48^. Within the BTE method, the carrier mobility *μ* in the relaxation time approximation can be express as Ref. 2 and 49:


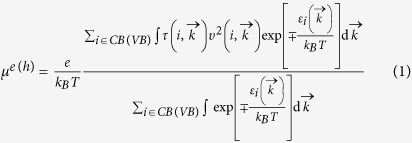


Where the minus (plus) sign is for electron (hole).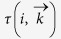 is the relaxation time, 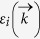 and 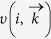 are band energy and the component of group velocity at 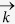 state of the *i-*th band, respectively. The summation of band was carried out over VB for hole and CB for electron. Furthermore, the integral of 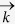 states is over the first Brillouin zone (BZ).

In order to obtain the mobility, three key quantities (
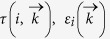
and 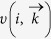) must be determined. The coherent wavelength of thermally activated electrons or holes at room temperature in inorganic semiconductors, which is much larger than their lattice constant, is close to that of acoustic phonon modes in the center of the first BZ. The electron−acoustic phonon coupling can be effectively calculated by the deformation potential (DP) theory proposed by Bardeen and Shockley^50^. So, the relaxation time 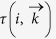
based on DP theory can be expressed as^2^,^48^


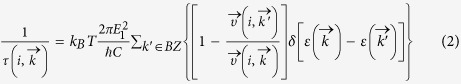


Here the delta function denotes that the scattering process is elastic and occurs between the band states with the same band index. 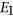
is the DP constant of the *i*-th band, and *C* is the elastic constant.

The band energy 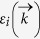 is calculated by the Vienna *ab-initio* simulation package (VASP)^51^. The 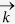 -mesh is chosen as 11 × 11 × 1 for electronic structures calculation and 61 × 61 × 1 for band eigenvalue calculation, which is fine enough to give converged relaxation time and mobility. The generalized gradient approximation (GGA)^52^ with the Perdew-Burke-Ernzerhof (PBE)^53^ exchange correlation function is used with the plane-wave cutoff energy set at 600 eV for all calculations. The criterion of convergence is that the residual forces are less than 0.001 eV/Å and the change of the total energy is less than 10^−7^  eV. The vacuum space between two adjacent sheets is set at least 15 Å to eliminate the interactive effect on each other.

The group velocity of electron and hole carriers can be obtained from the gradient of the band energy 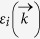 in 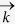-space, 
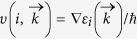
.

## Additional Information

**How to cite this article**: Xiao, J. *et al*. Theoretical predictions on the electronic structure and charge carrier mobility in 2D Phosphorus sheets. *Sci. Rep.* **5**, 09961; doi: 10.1038/srep09961 (2015).

## Figures and Tables

**Table 1:** The lattice length, distance of two P atom sub-layers (*d*), the energy per atom and average P-bond length in phosphorus sheets.

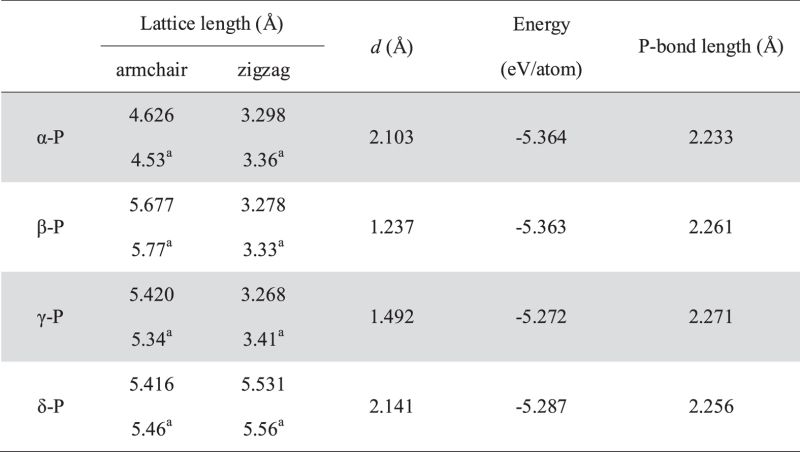

^a^Theory results from Ref. 21.

**Table 2:** The energy gap of phosphorus sheets:

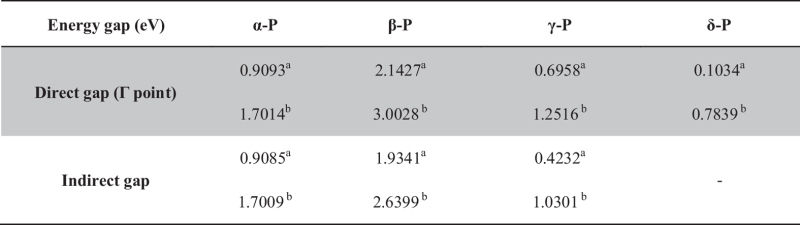

^a^is the PBE results;

^b^is the HSE06 results.

**Table 3:** The effective mass (m*) of carriers in phosphorus sheets.

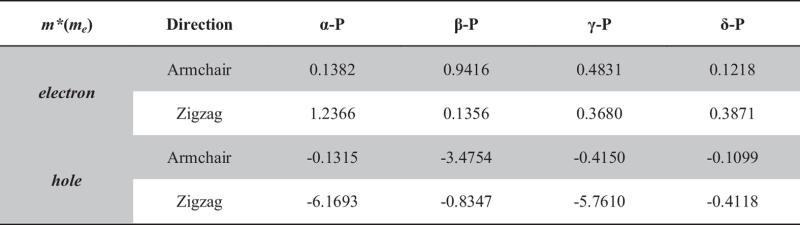

**Table 4:** Three-dimensional Young’s modulus (*C* ^*3D*^) of phosphorus sheets.

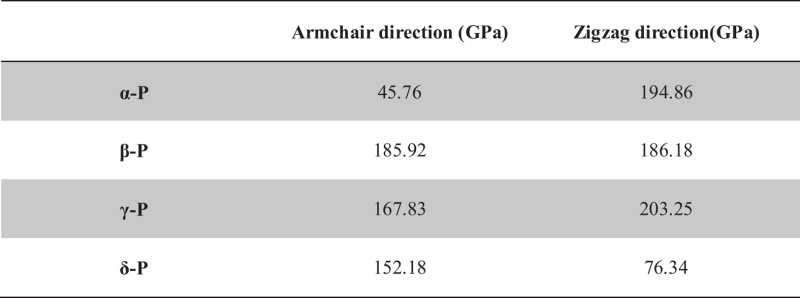

**Table 5:** The in-plane elastic constant (*C* ^*2D*^), deformation potential (*E*_*1*_), electron relaxation time (*τ*_*e*_), hole relaxation time (*τ*_*h*_), electron mobility (*μ*_*e*_) and hole mobility (*μ*_*h*_) in phosphorus sheets.

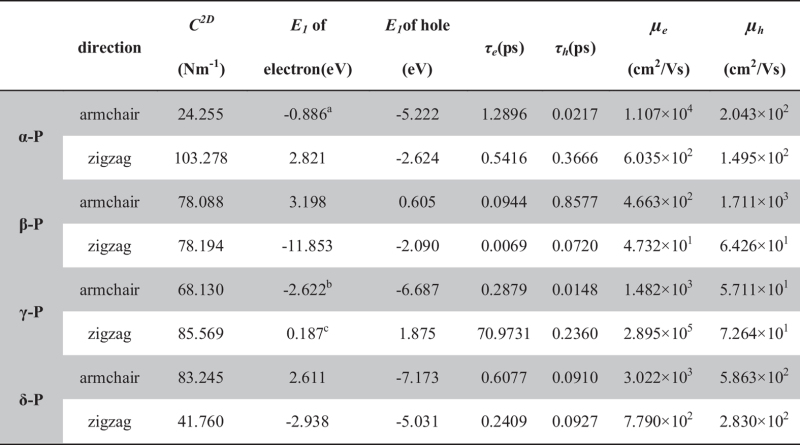

The temperature is 300K.The Standard Error is ^a^1.01%, ^b^1.78%, ^c^8.06% and others smaller than 1%.

**Table 6:** The percentage (%) of each orbit in the top of valance and the bottom of conduction.